# Steroid hormone signaling during development has a latent effect on adult male sexual behavior in the butterfly *Bicyclus anynana*

**DOI:** 10.1371/journal.pone.0174403

**Published:** 2017-03-22

**Authors:** Ashley Bear, Kathleen L. Prudic, Antónia Monteiro

**Affiliations:** 1 Department of Ecology and Evolutionary Biology, Yale University, New Haven, CT, United States of America; 2 Department of Entomology, University of Arizona, Tucson, AZ, United States of America; 3 Biological Sciences, National University of Singapore, Singapore; 4 Yale-NUS College, Singapore; University of Arkansas, UNITED STATES

## Abstract

It is well established that steroid hormones regulate sexual behavior in vertebrates via organizational and activational effects. However, whether the organizational/activational paradigm applies more broadly to the sexual behavior of other animals such as insects is not well established. Here we describe the hormonal regulation of a sexual behavior in the seasonally polyphenic butterfly *Bicyclus anynana* is consistent with the characteristics of an organizational effect. By measuring hormone titer levels, quantifying hormone receptor gene expression in the brain, and performing hormone manipulations, we demonstrate steroid hormone signaling early in pupal development has a latent effect on adult male sexual behavior in *B*. *anynana*. These findings suggest the organizational/activational paradigm may be more highly conserved across animal taxa than previously thought.

## Introduction

Hormones play a major role in regulating many sexual behaviors in vertebrates, but it is only recently that studies have demonstrated hormonal regulation of sexual behavior in insects [1–13]. In the vertebrate literature, hormonal effects on the parts of the brain that regulate sexual behavior (e.g., the neural substrates of sexual behavior) have often been defined as either organizational or activational. Organizational effects are traditionally defined as permanent modifications to the brain that occur during a critical period early in development and act to define certain characteristics of adult sexual behavior. In contrast, activational effects are defined as reversible effects of hormones on the brain which modify adult behavior by stimulating the neural substrates previously defined by organizational effects [1, 4, 6, 14]. Although it is now recognized that these definitions often represent extremes on a continuum of how hormones can affect sexual behavior, this paradigm remains prevalent in the literature as a powerful conceptual framework for describing how hormones regulate development [4, 6].

Recent studies on the hormonal regulation of insect sexual behavior have largely demonstrated activational effects of hormones during adulthood, while organizational effects on insect sexual behavior have rarely been described (but see select literature on caste systems in social insects [6]). Examples of such activational effects in insects include: the regulation of male attraction to female pheromones in the moth *Agrotis ipsilon* during adulthood by juvenile hormone (JH) and 20-hydroxyecdysone (20E) [7–9]; the regulation of male courtship behavior and female receptivity in the fruit fly *Drosophila melanogaster* during adulthood by JH [10, 11, 15]; the regulation of courtship behavior in the Carribean fruit fly *Anastrepha suspense* during adulthood by JH [13]; and the regulation of reproductive dominance behavior by the wasp *Polistes dominulus* during adulthood by JH [12].

*Bicyclus anynana* is a phenotypically plastic butterfly native to southern Africa that responds to cues from the environment to produce alternative forms [16]. The alternative seasonal forms of *B*. *anynana* are characterized by a suite of morphological, physiological and behavioral traits that are believed to serve adaptive functions in the seasonal environment that the adult butterfly will experience [17–24]. In particular, the dry season (DS) form has a cryptic color, with reduced ventral eyespots, which allows it to survive the dry season undetected by predators until the rains arrive and grasses for oviposition become available. The wet season (WS) form has large ventral eyespots that are important in deflecting the attacks of predators towards the wing margins [25], and are especially effective against invertebrate predators [26], which are abundant in the WS. Both forms, however, have different patterns of plasticity in their dorsal eyespots, which are used for sexual signaling [20, 27]. In particular, WS males use their large dorsal eyespots to court females, whereas in the DS, because males contribute to reproduction with a costly spermatophore, females use their large dorsal eyespots to court males [20]. DS males, thus, perform less courtship than WS males [20]. By mimicking the seasonal temperatures in the laboratory, we are able to study how temperature influences the development of each of these alternative traits.

Here we focus on examining the hormonal regulation of the plastic male sexual behavior in *B*. *anynana* [20, 21]. Previous research has shown males which develop at a cool temperature (17°C; typical of the DS) court females less frequently (exhibit a lower courtship rate) than males which develop at a warm temperature (27°C; typical of the WS) [20, 21]. Temperature influences the development of the courtship rate plasticity during a critical period that begins in the pupal stage [21], a period in development when the holometabolous insect brain undergoes a major developmental transition [28–30]. Because phenotypic plasticity in morphological traits is often mediated by environmentally-induced changes in hormonal signals, specifically 20-hydroxyecdysone (20E) [24, 31], we investigated whether, how, and when hormonal signaling variation during the pupal stage of development influenced courtship plasticity in adult males. By measuring hemolymph hormone levels, brain hormone receptor gene expression, and experimentally manipulating hormone levels throughout pupation, we explored whether the steroid hormone 20E could act as an organizational hormone for the regulation of an insect sexual behavior.

## Materials and methods

### Butterfly rearing conditions

The *Bicyclus anynana* lab colony was derived from 80 gravid females collected in Malawi in 1988 that have been reared in the lab ever since. A large number of eggs (~1000) were transferred to the University of Buffalo in 2002, which was later transferred to Yale University in 2006 and kept at around 200–300 breeding individuals each generation. Both seasonal forms are reared every year depending on experimental needs. Larvae were reared on young corn plants and adults were fed slices of banana. WS and DS forms were produced by rearing animals at 27°C, and 17°C, respectively. Humidity and light:dark cycles were kept constant at 80% and 12h:12h, respectively.

### Hormone titer measurements using Ultra Performance Liquid Chromatography and Mass Spectometry (UPLC/MS)

#### Hemolymph collection

A small puncture was made to the lateral posterior region of the fifth abdominal segment of individual pupae, at 2 pm, and 10 μl of hemolymph was collected using a pipet. Hemolymph collections were taken from WS and DS male pupae at four developmental time points: 14%, 30%, 50%, and 65% of pupal development (N per time point per seasonal form ranged from 3–8, with a median and mode of 4). Since insects are heterothermic and temperature has a direct effect on development time, developmental staging in this study depended on the percent of pupal development (calculated using measurements of total development time in each seasonal form). Thus, 14%, 30%, 50%, and 65% of pupal development corresponded to days 1, 2, 3, and 4 in the WS forms (total pupal developmental time ~ 6 days) and days 3, 6, 10 and 13 of DS forms (total pupal developmental time ~ 20 days). Hemolymph was also collected from DS males at 50% of pupal development that were previously injected with either 3 μl of 2000 pg/μl (6000 pg total) of 20E (Sigma-Aldrich^®^) or 3 μl of vehicle (10% EtOH and 90% saline) at 12 pm the same day (2 hours before collection) (N = 4 per treatment). Each sample was destructive so different individuals were measured at the developmental time points mentioned above. The time of day of hemolymph collection was held constant in order to avoid the confounding effects of daily fluctuations in hormone titers [32]. The 10 μl of hemolymph from each individual was then placed in a solvent solution of methanol/iso-octane (1:1, v/v). The hemolymph–solvent ratio was 1:10 (v/v). The mixture was vortexed for 20 seconds and then stored at -80°C until sample extraction. This sample preparation followed an established protocol [33].

#### Hormone extraction

We began by adding 900 μl of HPLC grade water to the 100 μl sample of 10 μl of hemolymph + 45 μl methanol + 45 μl iso-octane to make a 1000 μl volume in the sample vial and then vortexed the solution. We then used Waters^®^ Oasis HLB cartridges (1cc cartridge Part # 186000383) in an extraction manifold (Waters^®^ Part# WAT200677) to separate the hormone-containing methanol layer from the iso-octane layer. Before adding our hormone samples, we primed the HLB cartridges with 1000 μl methanol and then 1000 μl HPLC grade water. We then added the 1000 μl hormone-containing sample to the HLB cartridge, but did not collect the elution. Then, we added 1000 μl of HPLC grade water to the HLB cartridges and also did not collect the elution. We repeated this step three times to wash the cartridge. We then added 50 μl of methanol to the HLB cartridge to elute the hormone from the cartridge. The elution was placed at -20°C until hormone measurement.

#### Hormone titer measurements

20-Hydroxyecdysone was measured at the W.M. Keck Foundation Biotechnology Laboratory at Yale University using UPLC/MS [34–37]. This facility used a Perkin Elmer Flexar Ultra High Pressure Liquid Chromatography System coupled in-line to a 4000 Q-TrapLCMS/MS system. The hormones were separated utilizing an Agilent Technologies ZORBAX Eclipse XDB-C18 (3.0 x 100mm, 3.5 micron pore size) column (p.n. 961967–302) coupled to an analytical Phenomenex SecurityGuard trap (C18, 4 x 3.0mm) kept at 40°C. The samples were eluted at a flow rate of 500 μl/min using a methanol:water-based mobile phase which contained 0.1% formic acid. A blank injection of 100% methanol was run after each sample injection to ensure no carry over. Optimization of the differential potential (DP) and collision potential (CE) were carried out utilizing direct injection of 20E purchased (Sigma-Aldrich^®^ H5142). Source and gas conditions were optimized based on the standard direct injection runs. Next the standard was used determine the best gradient to use for the sample runs and calibrate specific transitions. We monitored three transitions, but only one transition (the one which provided the best detection response) was utilized in our quantitation measurements and reported (481.7 / 303.5). Caffeine was used as an external control to ensure instruments data acquisition stability and reproducibility for our specific gradient and instrument parameter settings. Data were acquired on the 4000 QTRAP instrument utilizing Analyst 1.5.2. and the collected raw data were processed utilizing Multiquant software (v. 2.0).

#### Hormone titer data analysis

We compared hormone titers from DS and WS butterflies at the equivalent stages of pupal development using t-tests, and the IBM^®^ SPSS^®^ Statistics Version 20 software. We used a Kolmogorov-Smirnov and Shapiro-Wilk test to assess normality in the data and Levene’s test to establish equality of variance between seasonal forms.

### Quantification of brain *Ecdysone receptor* (*EcR*) gene expression levels using real-time quantitative polymerase chain reaction (qPCR)

#### Sample collection

At 2 pm, pupal brains were dissected in ice-cold 1xPBS and then placed in RNAlater^®^ (Life Technologies). After a 24 hour incubation period at 4°C, the samples were stored at -80°C until RNA extraction. Four pupal brains were collected at each of four time points, 14%, 30%, 50%, and 65% of pupal development, per seasonal form.

#### RNA extraction

RNA was extracted using the RNeasy Plus Micro Kit (Qiagen^®^). On the day of extraction, each brain was homogenized using three RNase-free beads (#SSB14B 1.4mm Stainless Blend NEXT>>>ADVANCE^®^) that were placed into 1.5ml eppendorf tubes along with 350 ul Buffer RLT Plus. The tissue samples were disrupted using a Bullet Blender (NEXT>>>ADVANCE^®^) for three minutes at speed 7. The rest of the extraction protocol followed the instructions in the RNeasy Plus Micro Kit. The extracted RNA was then checked for quality using a ND1000 spectrophotometer (NanoDrop^®^ Technologies) and then immediately converted into cDNA using the High Capacity cDNA Reverse Transcription kit (Applied Biosystems^®^), after which time, the quality of the cDNA was also checked using a ND1000 spectrophotometer.

#### qPCR

In order to quantify relative levels of *EcR* gene expression in the *B*. *anynana* pupal brains, we used TaqMan primers and probes (Applied Biosystems^®^) targeting the predicted common sequence to all *EcR* isoforms and the single-copy “house keeping” *Ef1α* reference gene [38]. Two previous studies that evaluated reference genes for qPCR in *B*. *anynana*, showed that *Ef1α* was a stable reference, including for quantifying genes in brain tissue, with a threshold expression stability M value below 0.5 [39, 40]. EcR exists in several isoforms (distinct only in their N-terminal domains) that are produced by alternative splicing of the *EcR* gene. There are three known isoforms (EcR A, B1, and B2) in the dipteran *D*. *melanogaster* [30] and two (EcR A and B1) in the lepidopterans, *M*. *sexta* and *B*. *mori* [41, 42], however, the number of isoforms in *B*. *anynana* is not currently known. Here we targeted the region of EcR shared across all isoforms. Each 96-well plate contained the four biological replicates per temperature regime and three technical replicates. Each plate was run only with brain samples from butterflies at the corresponding percent of pupal development. We also included three technical replicates per gene of interest of a negative control, in which the probes, primers, and reagents were added to wells of the 96-well plate, but no cDNA was added. We set up 20 μl reactions that contained 1μl of 100 ng/μl of cDNA, 10 μl of the 2X TaqMan^®^ Fast Universal PCR Master Mix (Applied Biosystems^®^), 1 μl of 20X TaqMan^®^ Gene Expression Assay, and 8 μl of DNase free water. We used the following cycling conditions: pre-cycling step of 50°C for 2 min, 95°C for 20s for 1 cycle, and then 40 cycles of 95°C for 3 sec and 30 sec at 60°C. Primers for *Ef1α* were Fw: 5’-CCACCGATTTTGTAGACGTCTTGAA-3’; Rv: 5’-AGCACGTCCCACAGACAAG-3’. Primers for *EcR* common sequence were: Fw: 5’-CGAGCGAGGTGATGATGC-3’; Rv: 5’-AGCCCGCCTTGTTGTAGAT-3’.

#### qPCR data analysis

Following convention, only data from technical replicates with a standard deviation below 0.5 were used in the final analysis. Relative expression level of *EcR* was normalized by reference to *Ef1α* expression, and the 2-ΔCt method was used for comparative quantification [43]. These 2-ΔCt values were compared between WS and DS pupae for each developmental time point sampled (percent of pupal development) using t-tests, performed using the IBM^®^ SPSS^®^ Statisics Version 20 software. We used a Kolmogorov-Smirnov and Shapiro-Wilk to assess normality in the data and Levene’s test to establish equality of variance.

### Hormone manipulations

Male DS pupae were injected with 3 μl of 2000 pg/μl of 20E (6000 pg total) (cat. H5142 Sigma-Aldrich^®^) or vehicle at both 50% and 30% of pupal development. This concentration and injection volume were chosen after some optimization. We did not test the LD50 for edysone, instead we used the concentration that brought the hemolymph titers to a biologically relevant level found in WS forms, at the corresponding point in development, as indicated by the LC/MS measurements performed 2 hours after collection. The dosing took into account the volume of hemolymph in the average pupae, which we determined by extracting all the hemolymph from pupae with a pipette and then measuring the volume. Five mg of 20E powder was dissolved in 5 ml of 100% ethanol to make a 1 mg/ml solution. This stock solution was then further diluted in ethanol to produce a second stock of 20,000 pg/μl (10 μl of 1 mg/ml stock in 490 μl of ethanol). The stock solutions were kept at -20°C. Aliquots from the second stock were made fresh every week and were kept at 4°C. These consisted of 1 part of the 20,000 pg/μl 20E stock solution and 9 parts saline (20 μl of the 20,000 pg/μl stock in 180 pg/μl saline) (Life Technologies^®^), while the vehicle consisted of 1 part ethanol and 9 parts saline. The injections were done using a Hamilton syringe (10 μl 700 series hand fitted microliter syringe with a 33 gauge, 0.5 inch needle). The injection site was in the lateral left posterior region of the fifth abdominal segment [44].

### Quantification of courtship rate

On the day of eclosion (day 1), animals were separated by sex before 11 am to ensure virginity. All behavioral observations were performed on day 7, when five males from the same treatment group (untreated (WS or DS), 20E injected (DS only), or vehicle injected (WS or DS) were placed together with five untreated wild type WS form females (of the same age) in net cylindrical hanging cages (30 cm diameter, 40 cm height) at 5 pm (N ranged from 10 (all injection treatments) to 33 (Untreated controls). The sample size was higher in the untreated controls because these data acted as baseline data that were collected continuously throughout the duration of the study. The evening time of observation was chosen because *B*. *anynana* exhibits crepuscular courtship. The courtship observations were made at 27°C to control for the effect of temperature on adult behavior. The courtship rate (the number of times males courted during the observation period) was recorded during a 30-minute observation period. We accounted for unequal variances in courtship across treatments, determined using Levine’s test, by natural logarithm transformation of the data. T-tests were used to determine any significant effects of hormone versus vehicle injections on courtship rate and a 2-way ANOVA was conducted to assess the effect of vehicle injection versus no injection on courtship rate of both WS and DS butterflies.

## Results

### WS *B*. *anynana* males have higher titers of 20-hydroxyecdysone (20E) relative to DS males during the first 50% of pupal development but similar *EcR* gene expression in the brain

**Fig 1:**
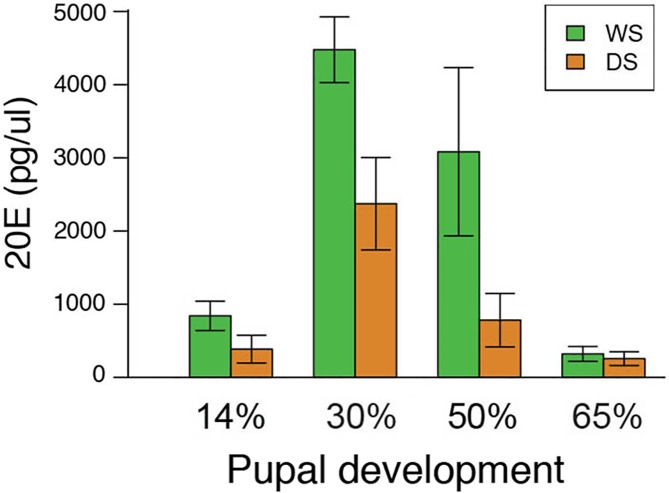
WS *B*. *anynana* males have higher titers of 20-hydroxyecdysone (20E) during early pupal development than DS males. T-test performed on hormone titer measurements show that there are significant differences in 20E titers in the hemolymph of *B*. *anynana* WS and DS male pupae at 14%, 30%, and 50% of pupal development but not at 65% pupal development. Error bars correspond to 95% confidence intervals for the means.

**Fig 2:**
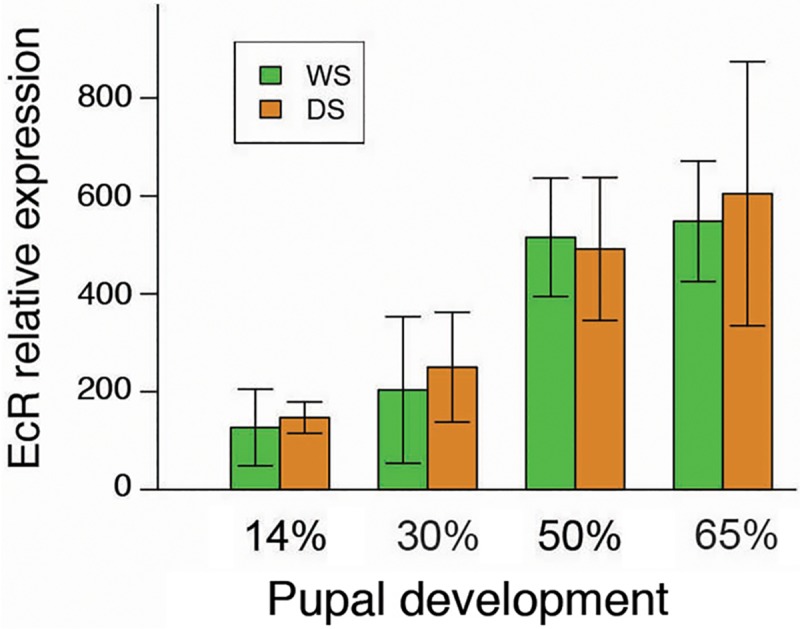
*Ecdysone receptor* expression in the brain is similar in pupal WS and DS B. anynana males. T-test performed on qPCR results show that there are no significant differences in *EcR* expression in the brains of *B*. *anynana* male pupae reared at WS and DS temperatures during 14%, 30%, 50%, and 65% of pupal development. Error bars correspond to 95% confidence intervals for the means.

We found significant differences in the 20E hormone titer between DS and WS pupa at 14% of development (t(6) = -3.235, p = 0.018), 30% of development (t(7) = -4.251, p = 0.004), and 50% of pupal development (t(10) = 2.678, p = 0.023), but no differences at 65% of development (t(6) = -0.907, p = 0.400) (Fig 1). In the first three time periods, WS males had higher titers relative to DS males. However, we did not find significant differences in relative *EcR* expression between DS and WS pupal brains at all points sampled during development (14% of development: t(6) = -.470, p = 0.663; 30% of development: t(6) = -0.648, p = 0.545; 50% of development: t(6) = -0.025, p = 0.814; and 65% of development (t(4) = -0.373, p = 0.728) (Fig 2).

### Artificially raising 20-hydroxyecdysone (20E) titers in DS *B*. *anynana* during 30% of pupal development significantly increases courtship rate

**Fig 3:**
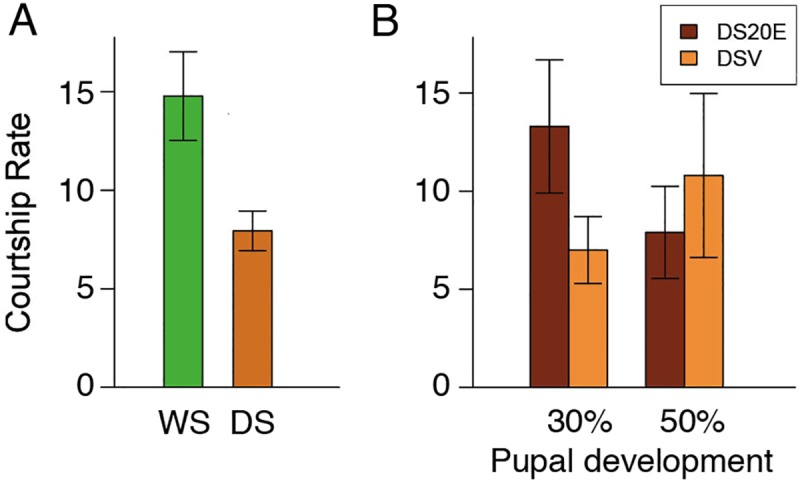
Raising 20E titers in DS males during pupal development significantly increases courtship rate. A) Butterflies reared at the WS typical temperature of 27°C have a significantly higher courtship rate than butterflies reared at the DS typical temperature of 17°C. B) Courtship rate of DS males injected with 6000 pg of 20E at 30% of pupal development (30%P) is significantly higher than the courtship rate of DS males injected with vehicle at the same time. There is no significant difference in the courtship rate of DS males injected with 20E at 50% of development (50%P) compared to DS males injected with vehicle at the same time (light brown). Error bars correspond to 95% confidence intervals for the means.

Untreated DS males exhibited significantly fewer courtship events per 30 minutes than untreated WS males (an average of 8 and 15, respectively; t(65) = 5.244, p<0.000) (Fig 3A), as documented in previous studies [20, 21]. This confirms that developmental rearing temperature influences courtship rate in adults, as males from the two temperature-treatments (WS versus DS) spent six days at a common temperature of 27°C prior to observation. Furthermore, the injection of vehicle, per se, did not alter the relative courtship rates in DS and WS males at either 30% pupal development (2-way ANOVA on courtship rate: rearing temperature: F_3,83_ = 23.764, p<0.001; treatment: F_3,83_ = 1.519, p = 0.221; interaction: F_3,83_ = 0.049, p = 0.825), or at 50% of pupal development (rearing temperature: F_3,83_ = 8.567, p = 0.004; treatment: F_3,83_ = 0.014, p = 0.906; interaction: F_3,83_ = 2.793, p = 0.098) (S1 Fig).

**Fig 4:**
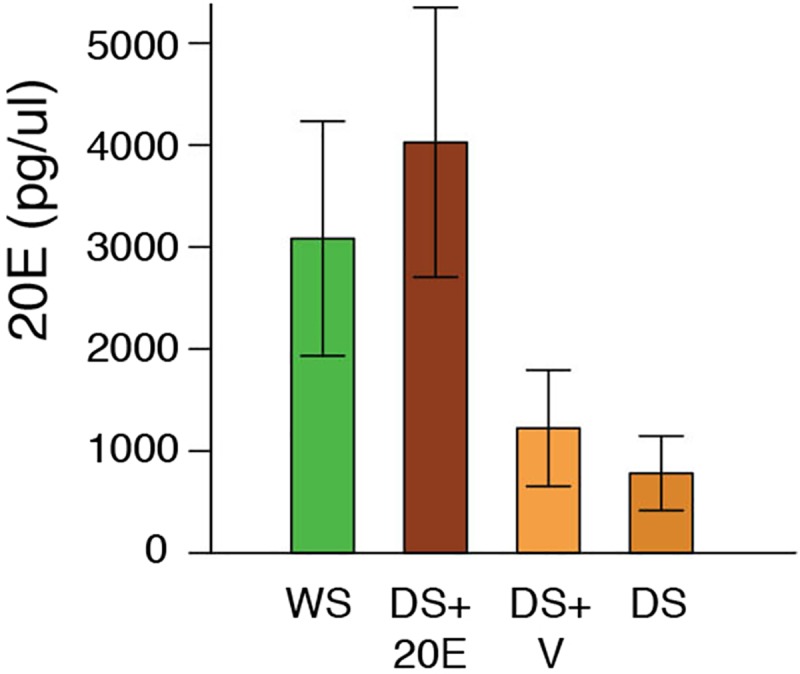
DS *B*. *anynana* males injected with 20E have higher titers than vehicle-injected animals. T-tests showed that DS *B*. *anynana* males injected with 6000 pg of 20E at 50% of pupal development showed significantly higher levels of 20E than DS males injected with vehicle at the same time. Error bars correspond to 95% confidence intervals for the means.

We injected DS males with either 20E or with a control vehicle, at both 30% and 50% of pupal development, and then scored their adult courtship rate. DS males injected with 20E at 30% of pupal development showed a significant, 62% increase in courtship rate relative to butterflies injected with vehicle at the same time during development (t(18) = -2.713, p = 0.014) (Fig 3B), but no such change was observed when 20E was injected at 50% pupal development (t(18) = -1.065, p = 0.301)(Fig 3B). DS males injected with 20E at 50% of pupal development, however, had significantly elevated 20E titers relative to animals injected with vehicle at 50% of pupal development (t(6) = 3.815, p = 0.009)(Fig 4), so the lack of a behavioral response is not due to a lack of a difference in the 20E titers caused by the injection.

## Discussion

Here we demonstrate that variation in hormone signaling during *B*. *anynana* development influences plasticity in an adult behavioral trait. By examining hormone titers, hormone receptor expression, and performing functional endocrine manipulations during the pupal stage of development, which was previously shown to be the beginning of the critical period for the courtship rate plasticity [21], we show that differences in hormone titers play a role in regulating the courtship rate plasticity in adult male *B*. *anynana*. We first found significant differences in 20E hormone titers between DS and WS males at 14%, 30%, and 50% of pupal development (Fig 1). At each of these developmental periods, WS pupae had higher titers than DS pupae. However, WS and DS males had equivalent *EcR* expression levels in the brain throughout the pupal stages investigated, suggesting no differences in sensitivity to 20E (Fig 2). Injections of 20E into DS male pupa, but not injections of vehicle, significantly elevated 20E titers post injection (Fig 4). Finally, injection of DS males with 20E at 30% of pupal development, but not at 50%, was sufficient to increase the courtship rate of DS butterflies (Fig 3B). Thus, the higher 20E titer at 30% of pupal development in WS males plays an important regulatory role in the adult courtship rate plasticity. We found no evidence to support the idea that the higher 20E titer at 50% of pupal development influences male courtship.

Differences in 20E levels between the DS and WS reared males examined in this study differ from those reported for females in a previous study [45]. In this study, female *B*. *anynana* reared at 19°C and 27°C, as well as intermediate temperatures, showed a shift in the timing of a 20E titer peak during pupation such that females reared at warmer temperatures exhibited an earlier 20E peak than those reared at cooler temperatures. Here we find that WS (27°C) males exhibit higher overall levels of 20E throughout pupation than DS (17°C) reared males. While the lower rearing temperatures between these two studies differed by 2°C, compromising comparisons across studies, taken together, these results suggest that *B*. *anynana* exhibits a sexually dimorphic endocrine response to temperature that should be further examined.

Although we focused our investigation on the effect of 20E levels in regulating plasticity in courtship rate, it is also possible that the hormone level differences we identified throughout pupal development could play a role in regulating other temperature sensitive, plastic traits exhibited by *B*. *anynana*. For example, wing patterns involved in sexual signaling [20, 23], eye morphology and physiology [46, 47], and sex pheromone production [22] could all respond to changes in 20E hormone titers. This type of integration could explain how sex pheromones, sexual ornaments, organs used to visualize such ornaments, and sexual behaviors could co-evolve in *B*. *anynana*. Investigations into how 20E signaling during mid pupal development influences sexual ornaments on *B*. *anynana* wings are currently underway.

These results are consistent with an organizational effect of hormones on adult behavior for several reasons. First, differences in 20E titers at 30% of pupal development have a significant effect on a behavior that is exhibited in adulthood. The extreme latency of this effect is compatible with the characteristics of organizational effects in vertebrates [1, 4, 6]. Second, the hormonal regulation of the adult courtship rate occurs during a critical period when the brain is undergoing major development [48–50]. Third, the hormone implicated in this case, 20E, is a steroid hormone that is known to play a major role in coordinating the development of the brain during pupal development [49, 50], suggesting that 20E signaling in *B*. *anynana* at 30% of pupal development may regulate the adult courtship phenotype by inducing lasting morphological changes on the brain, another characteristic of an organizational effect [1, 4, 6]. Finally, the temperature experienced by the adult butterflies observed in this study remained constant (27°C) across treatments, while the courtship rate plasticity persisted, this indicates permanence of the effect of a hormone on behavior, which is typical of an organizational effect.

While the results of this study suggest that 20E may have an organizational effect on a sexual behavior in *B*. *anynana*, a closer examination into whether 20E induces morphological changes in the *B*. *anynana* pupal brain would be required to support this conclusion. We are currently pursuing an answer to this question by first investigating how temperature and 20E levels affect gene expression in the developing *B*. *anynana* brain using RNA-Seq [51, 52]. By identifying genes that are differentially expressed in response to WS- and DS-typical temperatures and/or 20E levels, and then localizing their expression domain via in situ hybridizations, we hope to be able to resolve whether there are differences in neural morphology between animals reared at the WS and DS temperatures and/or exposed to WS and DS typical 20E titers.

Although the results of the present study may be indicative of an organizational effect of 20E on the *B*. *anynana* brain and behavior, alternative hypotheses should also be considered. For example, it is possible that the 20E titer at 30% of pupal development does not have a direct effect on male brain development, but rather drives the alternative development of a male physiological or morphological trait that might influence the behavior of adult female butterflies in a way that promotes or deters courtship by the males. Examples could include season specific differences in the brightness of UV reflective eyespots on the wings, which are known to play a role in courtship signaling [20, 53], or season specific differences in the pheromones that male *B*. *anynana* are known to emit from specialized structures on the wing [22, 54]. Alternatively, 20E titer levels at 30% of pupal development might influence the development of the glands that produce 20E during adulthood, such as the gonads [55], resulting in activational effects of 20E, or other hormones, on the brain during adulthood. However, it is also possible, and indeed would be consistent with the original conception of the organizational/activational paradigm, that temperature-mediated 20E signaling has both an early developmental influence on the morphology of the *B*. *anynana* brain (organizational) that is later activated (activational) by a rearing temperature specific profile of hormone secretion during adulthood. Future research that examines the effect of hormones in adult *B*. *anynana* will address these alternative hypotheses and will act to further clarify how 20E signaling during pupal development modifies adult courtship behavior.

## Conclusion

Our results demonstrate that a steroid hormone acting during development can have a latent effect on adult sexual behavior in an insect. These findings are consistent with the characteristics of an organizational effect, a mechanism of hormone action that has rarely been documented in the context of insect sexual behavior. These findings raise questions about whether the organizational/activation paradigm of hormonal regulation of sexual behavior applies more broadly across the animal tree of life than previously thought.

## Supporting information

S1 Fig Number of courtship events observed in a 30 minute period for all the control and experimental groups evaluated in the current study. The experimental groups were injected with vehicle or 20E at 30% (top graph) and 50% (bottom graph) of pupal development.
(PDF)
