# Supplementary figures and images for: Steroid hormone signaling during development has a latent effect on adult male sexual behavior in the butterfly Bicyclus anynana
Source: PLoS One. 2017 Mar 22;12(3):e0174403. doi: 10.1371/journal.pone.0174403 (PMC5362226; doi:10.1371/journal.pone.0174403)

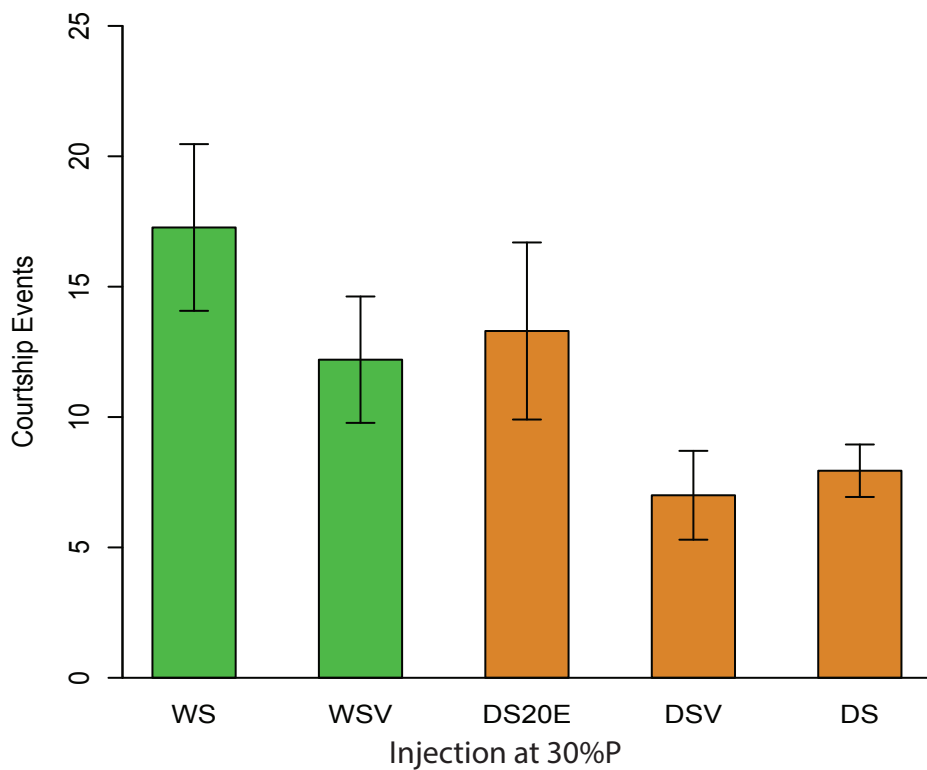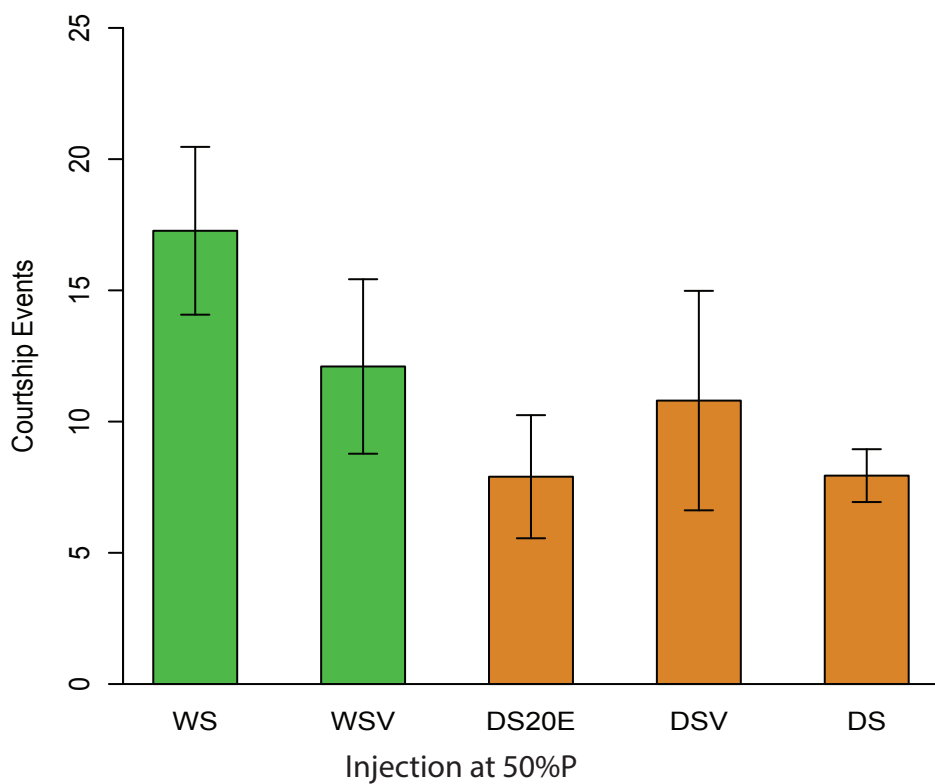

Supplement: S1 Fig — The experimental groups were injected with vehicle or 20E at 30% (top graph) and 50% (bottom graph) of pupal development. (PDF) [file pone.0174403.s001.pdf]
